# Mr. Higginbottom on Lunar Caustic in Inflammation

**Published:** 1826-07

**Authors:** 


### POWERS OF LUNAR CAUSTIC IN ARRESTING INFLAMMATION.*

Mr. Higginbottom, whom our readers will no doubt recollect, has laid before the public some more cases of local inflammation, successfully treated by his favorite remedy, the nitrate of silver.

A servant girl, æt. 16, complained of pain and swelling attended with inflammation on the fore part of the knee. The skin was sore and hard to the touch; pulse frequent and feverish—she was accustomed to kneel in washing, &c.—an emetic and purgative were given and the lunar caustic applied all over and beyond the inflamed part. Two days afterwards the swelling had much subsided; the tenderness was gone. In a few days the girl was well.

This case was doubtless one of incipient irritation and inflammation of the bursa mucosa. We have seen similar instances, where leeches, lotions, rollers, &c. have been applied, and, at the end of some weeks the patient has been worse than at first.

A servant maid, æt. 24, pricked the middle finger of the right hand. It became swollen and extremely painful, as well as the back part and palm of the hand. On opening the wound with the lancet a little pus escaped. The caustic was well applied to the wound and also to the inflamed parts of the finger, and hand. An emetic and purgative were given and the hand kept in a sling. The symptoms had disappeared in the course of two days, and she quickly recovered.

A girl, æt. 20, wounded the first joint of the fore-finger with the bone of a hare. In a few days afterwards the finger became swollen, painful, and affected with erysipelatous inflammation reaching to the back of the hand, and bordered by a ring of a more vivid colour. Caustic ap-

* Med. and Phys. Journal for May, 1826

plied as usual. In two days time the swelling had become puffy, and the pain disappeared. The hand, in a few days, was quite well.

A country woman had several fingers and great part of one hand much swollen with considerable pain; some fever: tongue white; a degree of pain in the axilla. An emetic and a purgative with calomel were given, and the lunar caustic applied. Three days afterwards the patient was free from complaint.

We must acknowledge that we have seen cases apparently quite as simple as several of these end in a very different manner. Some have run on to suppuration, sloughing, and ended in amputation. Other patients have had the luck of being on the list for a few weeks, perhaps months, and escaped at last with a stiff hand or knee as the case might be. Few, very few, have got off so cheaply as our author's patients. Mr. Higginbottom recommends the inducing of an eschar over the seat of an internal disease or inflammation. We think this mode of employing the caustic will hardly be of such service, for here the cautery can operate but as an issue, having no local nor specific action in checking the inflammation.

